# Comparative analysis of in vitro accelerated glistening formation in foldable hydrophobic intraocular lenses

**DOI:** 10.1007/s10792-021-01870-6

**Published:** 2021-05-19

**Authors:** Tamer Tandogan, Gerd U. Auffarth, Chul Young Choi, Hyeck-Soo Son, Ramin Khoramnia

**Affiliations:** 1 The David J. Apple International Laboratory for Ocular Pathology and International Vision Correction Research Centre (IVCRC), Department of Ophthalmology, University of Heidelberg, INF 400, 69120 Heidelberg, Germany; 2 Department of Ophthalmology, Kangbuk Samsung Hospital, Sungkyunkwan University School of Medicine, Seoul, Korea

**Keywords:** Intraocular lens, Laboratory analysis, Glistenings

## Abstract

**Purpose:**

To analyse and compare the propensity to form glistenings in 4 different types of hydrophobic acrylic intraocular lenses (IOLs): Alcon AcrySof ® MA60AC, HOYA iSert® PC-60AD, Bausch&Lomb enVista, and Kowa Avansee™ PU6A.

**Methods:**

We used an accelerated laboratory method to create glistenings. IOLs were first immersed in saline at 45 °C for 24 h and then at 37 °C for 2.5 h. Microvacuole (MV) density and size were documented and calculated using an image analysis program.

**Results:**

Median density of glistenings [MV/mm^2^] for Alcon AcrySof ® MA60AC was 623 (range 507–804), for HOYA iSert® PC-60AD 1358 (range 684–2699), for Bausch&Lomb enVista 2 (range 1–2), and for Kowa Avansee™ PU6A 1 (range 1–4). The prevailing MV size was: 0–5 µm for Hoya IOLs, 5–10 µm for Alcon IOLs, 20–50 µm for Bausch&Lomb IOLs, and 5–50 µm for Kowa IOLs.

**Conclusions:**

Glistenings could be induced in all studied IOLs using the accelerated laboratory method. The Alcon AcrySof ® MA60AC and HOYA iSert® PC-60AD IOLs showed MV of high density, while the glistenings in the Hoya IOLs were smaller in size compared to the Alcon IOLs. The MV density was minimal in the Bausch&Lomb enVista and Kowa Avansee™ PU6A IOLs. The propensity of the Alcon AcrySof ® MA60AC IOLs to form glistenings in vitro correlated with the findings of clinical results that are already published.

## Introduction

Glistenings describe small, refractive microvacuoles that arise within the biomaterial of intraocular lenses (IOLs) [1–4]. Studies have reported on their clinical significance as they may deteriorate the patients’ visual acuity and contrast sensitivity [5–10].

Since 1984, such glistening formation has been described in different IOL materials such as rigid hydrophobic polymethyl methacrylate, foldable silicone, and foldable hydrophobic acrylate. According to an ASCRS survey, the foldable hydrophobic acrylic IOLs were currently the most commonly implanted IOLs [11]. The incidence of glistening formation and its severity are reported to be the highest with foldable hydrophobic acrylic materials [7, 12, 13].

The simulation of glistening formation in a laboratory setting can be achieved via an accelerated ageing process. Such in vitro studies are considered to give valuable information about the tendency of a given material to form glistenings [12, 14–20].

Several hydrophobic IOL materials have already been analysed under laboratory conditions to compare their tendency to create glistenings [14–20]. The Bausch&Lomb enVista IOL is reported to be free of glistenings even 2 years after implantation, giving this IOL the label as being “glistening-free” [21]. Another new generation hydrophobic foldable IOL (Kowa Avansee™) has been introduced which purportedly does not have a propensity to develop any glistenings.

In this study, we compared the glistening formation of this IOL of Kowa (Kowa Avansee™ PU6A) to the FDA-approved glistening-free Bausch&Lomb enVista IOL as well as to two widely used hydrophobic IOL materials, namely the materials in the hydrophobic HOYA iSert® PC-60AD IOL and the Alcon AcrySof ® MA60AC IOL, which have been extensively studied for their glistening formation previously [14–20, 22].

## Material and methods

Five IOLs of each type were included in this comparative laboratory study: Alcon AcrySof ® MA60AC, HOYA iSert® PC-60AD, Kowa Avansee™ PU6KA and Bausch&Lomb enVista. All IOLs had a dioptric power of + 20.0 D and were composed of clear (not yellow tinted) hydrophobic acrylic material with an integrated UV inhibitor. Table 1 shows the material composition of the IOLs examined. All lenses analysed in the current study were newly manufactured at the time and suitable for implantation.

**Table 1:** Material composition of the studied intraocular lenses

	Material composition
Alcon AcrySof® MA60AC	Copolymer of phenylethyl acrylate and phenylethyl methacrylate, cross-linked with butanediol diacrylate
Hoya iSert® PC-60AD	Cross-linked copolymer of phenylethyl methacrylate and n-butyl acrylate, fluoroalkyl methacrylate
Bausch&Lomb enVista	Hydrated to equilibrium water content and packaged in a physiological saline solution to prevent glistening formation Designed with a high water content to enhance exibility and foldability
Kowa Avansee™ AU6KA	Hydrophobic soft acrylic (UV-absorbing acrylic resin; natural type also contains proprietary blue light filtering)

Using the now widely established methodology described by Thomes and Callaghan [14], glistenings were generated as aqueous-filled microvacuoles (MV) by using an accelerated laboratory ageing method in all IOLs. The IOLs were placed in bottles filled with saline solution. These bottles were placed in an oven set at 45 °C ± 1 °C. After 24 h, the lenses were moved to a 37 °C ± 1 °C water bath where they remained for another 2.5 h. Afterwards, the samples were analysed. The equipment for IOL analysis consisted of a microscope (MEIJI EMZ-TR8) with a heated stage, a CCD camera, a computer, and an image analysing software (i-Solution). The IOLs were observed visually under the light microscope and analysed regarding size and density of the MV at a specified temperature. The heated stage allows maintenance of the temperature of the lens at 37 °C during imaging which helps to maintain MV size and density during imaging.

**Fig. 1:**
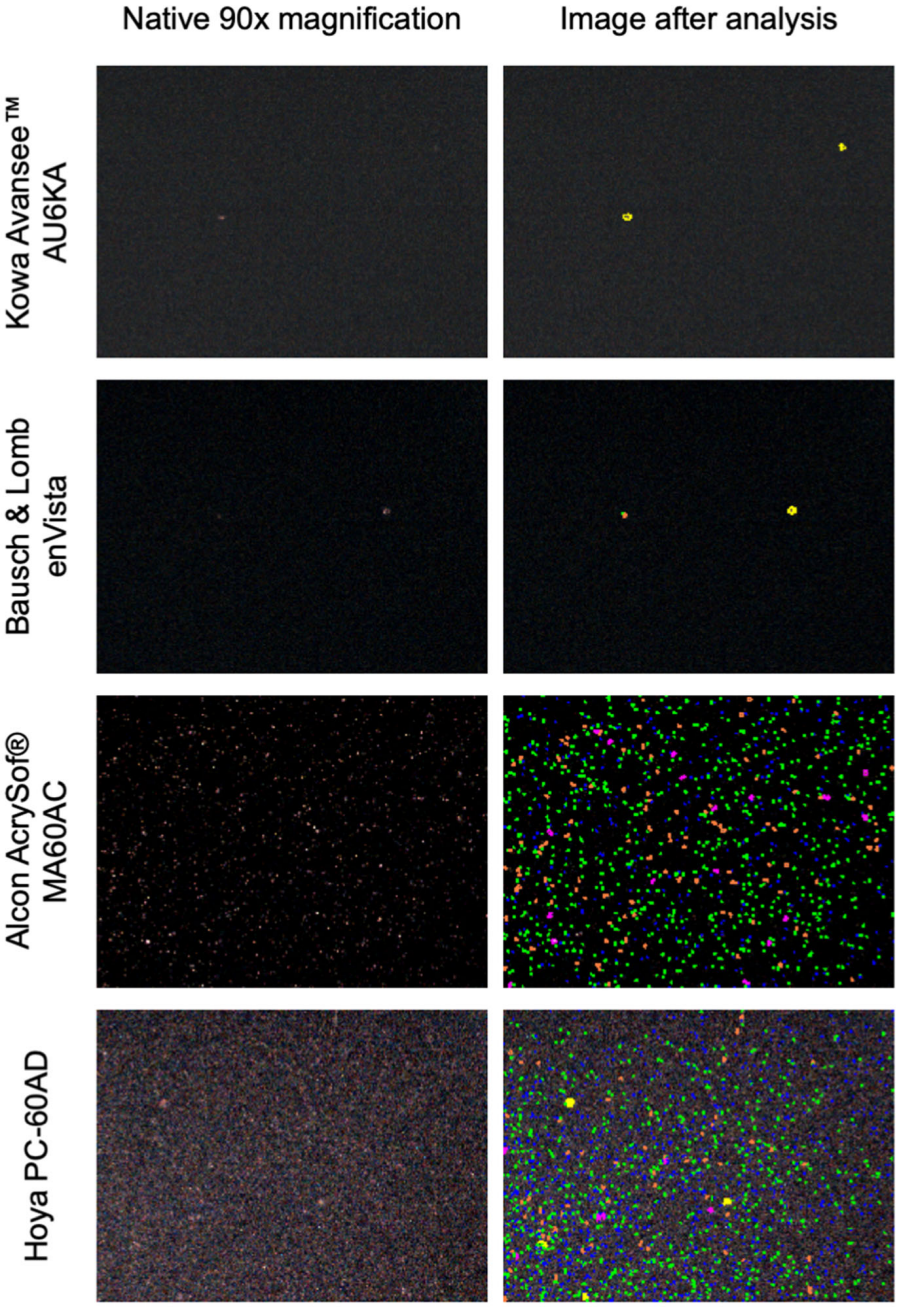
Detection of glistenings from the native picture (90-fold magnification, left) using the imaging software (*i*-Solution) (right)

The entire lens was scanned and the region of maximum density (central or paracentral and at the right focal plane) was captured for analysis. The resultant images were examined using an image analysis program (Fig. 1). The data acquired from these images were used to measure the density and size of the MV (units: number of microvacuoles per square millimetre [MVs/mm^2^]). Induced MVs were then classified depending on their size (Class 1: 0–5 µm, Class 2: 5–10 µm, Class 3: 10–15 µm, Class 4: 15–20 µm, Class 5: 20–50 µm, Class 6: 50–100 µm, No Class: > 100 µm) and density [15].

## Results

**Fig. 2:**
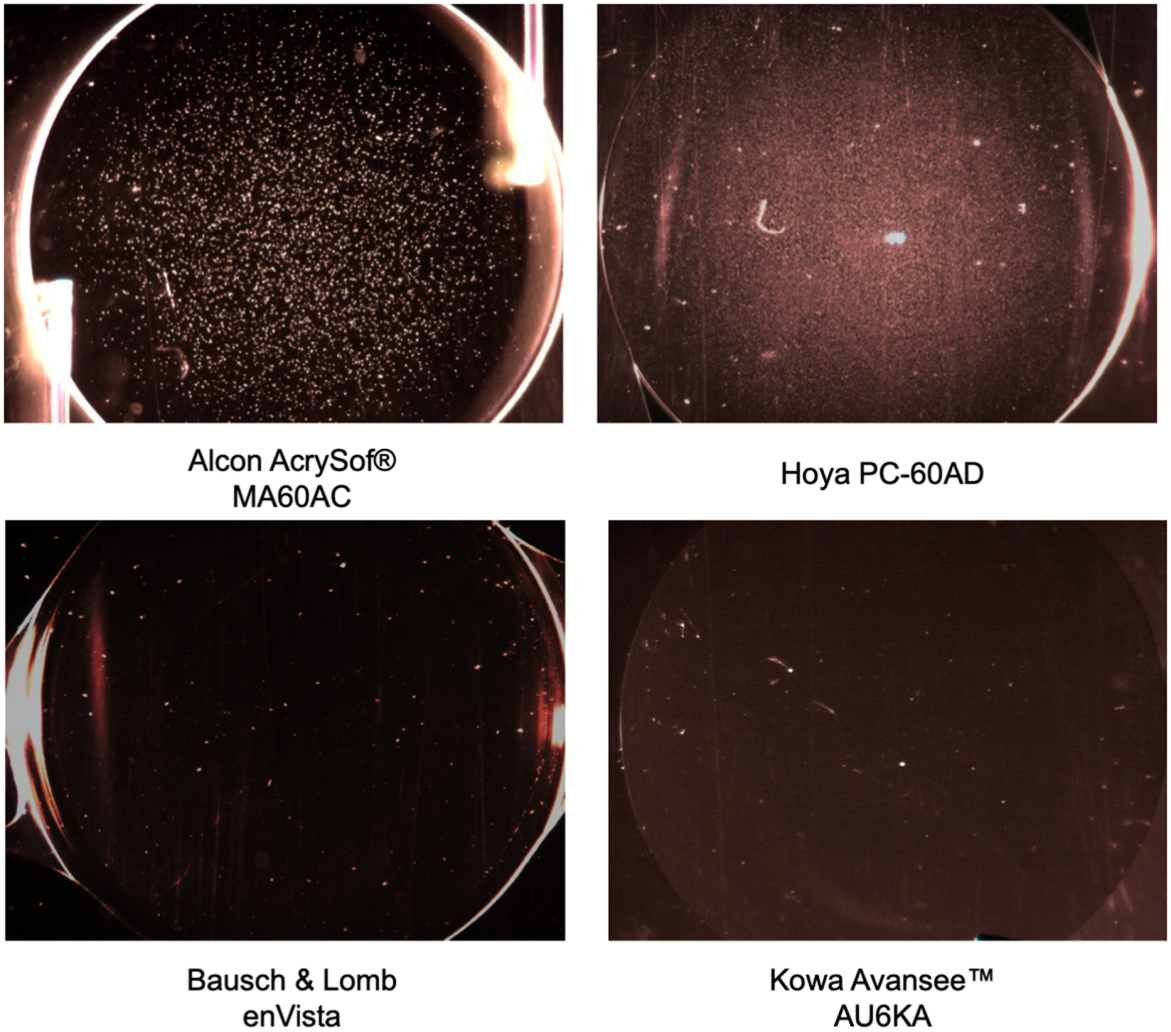
Example of all 4 IOLs (14-fold magnification)

**Fig. 3:**
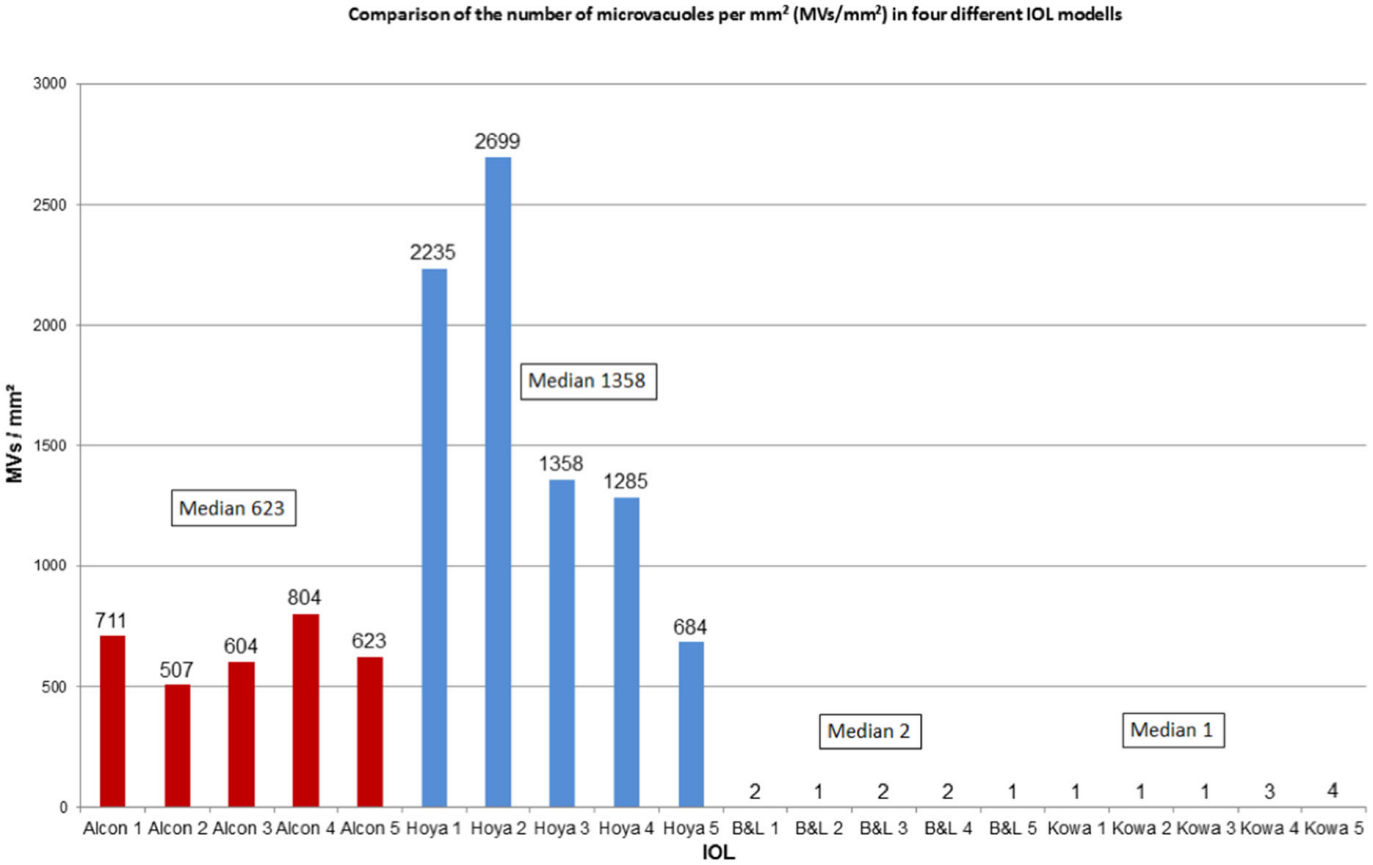
Microvacuoles per mm^2^: Data of all IOLs

Glistenings were detectable in all lenses after the accelerated microvacuole test method (Fig. 2). However, differences were found between the different types of IOLs (Fig. 3). The HOYA iSert® PC-60AD and Alcon AcrySof ® MA60AC IOLs showed rather high number of glistenings, while the Bausch&Lomb enVista and Kowa Avansee™ PU6KA materials were almost completely free of glistenings (Table 2).

**Table 2:** Scoring of the microvacuole density according to Miyata et al. [15]

	Microvacuoles/mm^2^	Grade according to Miyata et al. (Scale from 0 to 3)
Alcon AcrySof® MA60AC	> 200	3
Hoya iSert® PC-60AD	> 200	3
Bausch&Lomb enVista	< 5	0
Kowa Avansee™ AU6KA	< 5	0

The difference in the MV density was statistically significant and the density was higher in HOYA iSert® PC-60AD lenses compared to Alcon AcrySof ® MA60AC lenses. Both lenses showed higher MV density than the Bausch&Lomb enVista and Kowa Avansee™ PU6KA IOLs. There was no statistically significant difference in the MV density between the Bausch&Lomb enVista and Kowa Avansee™ PU6KA IOLs (*p* < 0.05, Mann–Whitney *U* test) (Table 3).

**Table 3:** Statistical analysis of the microvacuole density

	Alcon AcrySof® MA60AC	HOYA iSert® PC-60AD	Bausch&Lomb enVista	KOWA Avansee™ AU6KA
Alcon AcrySof® MA60AC		0.032*	0.009*	0.009*
Hoya iSert® PC-60AD	0.032*		0.009*	0.009*
Bausch&Lomb enVista	0.009*	0.009*		0.914
Kowa Avansee™ AU6KA	0.009*	0.009*	0.914	

^*^Statistically significant difference (*p* < 0.05, Mann–Whitney *U* test)

In HOYA iSert® PC-60AD IOLs, the prevailing MV size was Class 1. The Alcon AcrySof ® MA60AC IOLs and the Bausch&Lomb enVista IOLs showed prevailing MV sizes of Class 2 and 5, respectively. The Kowa Avansee™ PU6KA IOLs showed a balanced distribution of MV size between Classes 2 and 5 (Table 4).

**Table 4:** Distribution of the microvacuole size in all studied lenses

Mean microvacuole size (MV/mm^2^) and distribution							
	Class 1 0–5 µm	Class 2 5–10 µm	Class 3 10–15 µm	Class 4 15–20 µm	Class 5 20–50 µm	Class 6 50–100 µm	No Class > 100 µm
Hoya iSert® PC-60AD	1785	831.4	141	35.2	15.6	0.6	0
Alcon AcrySof® MA60AC	412.8	604.8	74.4	11.8	0.8	0	0
Bausch&Lomb enVista	0	0.4	0.8	1	1.8	0.2	0
Kowa Avansee™ AU6KA	0	1	0.6	0.6	0.8	0.2	0

The MVs were predominantly located in the midperipheral region of the optic in the Bausch&Lomb and Kowa IOLs. In contrast, the MVs were more densely distributed in the centre in Hoya and Alcon IOLs.

## Discussion

The glistening phenomenon is clinically important because of its possible effect on vision. Some studies suggested that severe glistenings could mildly decrease contrast sensitivity and visual acuity [8, 11, 23, 24].

Glistenings describe fluid-filled microvacuoles that form within the matrix of the IOL material when it is exposed to an aqueous environment [14]. Since there is a significant difference in the refractive index of water droplets (*n* = 1.33) and the bulk polymer (*n* = 1.55), the light is refracted and scattered at the water–polymer interfaces which are then visible as “glistenings” [16]. Among others, they have been mostly reported in hydrophobic acrylic IOLs [7, 12, 13].

We performed an IOL optical purity assessment by using temperature changes to accelerate the development of glistenings and quantified the density of the glistenings in different hydrophobic acrylic IOLs. Our results have shown that the Bausch&Lomb IOLs, which are claimed to be glistening-free, as well as the Kowa IOLs showed only a negligible number of glistenings. In terms of MV size and density, the two IOLs did not show any statistically significant differences. Previously, two clinical studies reported that no glistenings were observed at any postoperative visit in the Bausch&Lomb IOLs [25, 26]. In our experiment, no IOL model was completely free of glistenings. However, the numbers were very close to zero in the Bausch&Lomb and Kowa material.

Our results have also shown that the Alcon and the Hoya IOLs had a higher MV density than the Bausch&Lomb and the Kowa IOLs. The Hoya IOL showed the smallest MV size compared to the other studied IOLs.

Regarding the localization of the MVs within the IOL optic, the Alcon and the Hoya IOLs showed a higher MV density at the central part of the optic, while the Kowa and the Bausch&Lomb IOLs developed glistenings more at the mid-peripheral area of the optic, which may reduce the risk of a clinical impact in-vivo.

According to the glistening scoring scale described by Miyata et al. [15], the Kowa and the Bausch&Lomb IOLs were classified as Grade 0, while the Hoya and Alcon IOLs were classified as Grade 3. These results correlated with those of a study by Kawai et al. in which the Alcon and Hoya IOLs showed glistenings after an accelerated glistening method, while the Kowa Avansee™ and the hydrophilic acrylic Bausch&Lomb Hydroview IOLs did not [18]. However, the authors graded the glistenings distribution based only on a qualitative assessment in contrast to our qualitative, quantitative, and morphological assessment.

The tendency of the AcrySof material to form glistenings under laboratory conditions correlate well with the findings of clinical results which have already been published [8, 9]. While there do exist differences in the number of glistenings observed for each IOL in different in vitro studies, it is important to note that there are generally variations of glistening development and size even within the same IOL model. While the HOYA IOLs also showed a strong tendency to generate glistenings that were smaller in size compared to those of the Alcon IOL and more diffusely distributed, there are no clinical data published to date regarding glistenings observed *in-vivo* with HOYA iSert® PC-60AD IOLs.

This may, in part, be due to the fact that the present study did not simulate the temperature fluctuations in the human eye. Although glistening formation induced *in vitro* by alteration of the temperature can produce morphological aspects that in general appear exaggerated in comparison to the clinical situation, *in vitro* studies are considered suitable for laboratory investigation [12, 18, 27]. It is uncertain whether glistenings produced with laboratory methods arise due to the same mechanism or are of the same kind as glistenings observed in patients [14]. The rate of the temperature fluctuations seems to have a significant impact on the extent of glistening formation. Although in vitro analysis may provide an assessment of the tendency of a material to form glistenings, the correlation between in vitro test results and in-vivo observations remains unclear. While some studies showed that there is no impact of glistenings on the visual quality [12, 28, 29], others reported deterioration of visual acuity, contrast sensitivity, and an increase in straylight [5, 6, 23, 30, 31]. Furthermore, the osmolarity of the fluid around the IOL may play a role as do comorbidities of the patients such as diabetes mellitus. Inflammations or an interrupted blood–aqueous barrier may also influence the outcome in every patient [1–3].

It has to be emphasized that the problem of glistenings seems to have been fully resolved by the new Clareon material introduced by Alcon, which was shown to be glistening-free in preclinical in vitro studies [32]. However, the material evaluated here is also still used, even in more modern IOL optics [33, 34]. Hoya has also already introduced a new glistening-free material called Vivinex [35, 36].

Our results permit a comparison between different IOL models in their tendency to produce glistenings, and we demonstrated that this tendency can be significantly lowered in IOLs composed of modern IOL materials.

## Data Availability

The datasets used and/or analysed during the current study are available from the corresponding author on reasonable request.
